# Disentangling the age-related manner in the associations between gut microbiome and women’s health: a multi-cohort microbiome study

**DOI:** 10.1080/19490976.2023.2290320

**Published:** 2023-12-07

**Authors:** Chao Dong, Quanquan Guan, Wei Xu, Xiaochen Zhang, Bowen Jin, Shumin Yu, Xiaoyu Xu, Yankai Xia

**Affiliations:** a State Key Laboratory of Reproductive Medicine and Offspring Health, School of Public Health, Nanjing Medical University, Nanjing, China; b Key Laboratory of Modern Toxicology of Ministry of Education, School of Public Health, Nanjing Medical University, Nanjing, China; c Department of Gynecology, The First Affiliated Hospital of Nanjing Medical University, Nanjing, China

**Keywords:** Women’s health, gut microbiome, age, age-specific species markers, inflammatory bowel disease

## Abstract

Women’s health encompasses life-course healthcare, and mounting evidence emphasizes the pivotal contribution of gut microbiota. Therefore, understanding the temporal dynamics of gut microbiota and how age influences disease-gut microbiota associations is essential for improving women’s health. By analyzing metagenomic data from 3625 healthy women, we revealed significant effects of age on gut microbiota and age-dependent patterns in microbial features, such as relative abundance, Shannon index, and microbial network properties. Additionally, declining trends in the predictive accuracy of gut microbiota for age groups were shown using iterative sub-sampling based random forest (ISSRF) model. Age-specific species markers were also identified, many of which were shared across age groups. To investigate the influence of age on disease-gut microbiota associations, metagenomic data from 681 women with various disease conditions and 491 matched healthy controls were collected. A substantial proportion of species markers for inflammatory bowel disease (IBD), type 2 diabetes (T2D), atherosclerotic cardiovascular disease (ACVD), and impaired glucose tolerance (IGT) differed in relative abundance across age groups, and were also age-specific species markers. Besides, the microbiota-based probabilities of IBD and ACVD were positively correlated with age. Furthermore, the age specificity of disease-gut microbiota associations was explored using the ISSRF model. Associations between IBD and gut microbiota were age-specific, with reduced stability of disease species markers in childhood and adolescence, possibly due to decrease in the effect size between patients and controls. Our findings provided valuable insights into promoting healthy aging and developing personalized healthcare strategies for women.

## Introduction

Women’s health encompasses the physical, mental, and social well-being of women, which has seen substantial progress in recent decades.^1^ The worldwide priority to women’s health has shifted from a narrow focus on maternal and child health to a more comprehensive perspective. Therefore, there is a call to redefine and expand the global agenda for women’s health, ensuring sufficient resource allocation across all domains, and embracing targeted life-course approaches.^2,3^ Influential factors of women’s health are multifaceted and include biological, social, environmental, and behavioral determinants.^4,5^ Recent studies have shed light on the significant role of gut microbiota in various aspects of women’s health, including reproductive health,^6^ obesity,^7^ mental health disorders,^8^ and autoimmune diseases.^9^ Gut microbiota exerts its influence through diverse mechanisms, such as immune modulation,^10^ hormone and nutrient metabolism,^11^ production of bioactive metabolites,^10^ and regulation of gut barrier integrity.^12^ Consequently, exploring the impacts of gut microbiota on women’s health is a burgeoning field, which holds promise for future preventive and therapeutic interventions.

Host factors, such as sex, diet, lifestyle, and medication use, play a crucial role in shaping the composition and function of gut microbiota.^13^ Within the framework of life-course approaches on human health, there is a growing scholarly focus on investigating the influence of age on gut microbiota. To date, mounting population-based studies have consistently identified age as a prominent determinant of gut microbiota.^14^ The phylogenetic compositions of gut microbiota gradually shift toward a mature configuration during the first three years after birth, and diversity measurements exhibit an upward trend across the life course.^15^ Compared to younger adults, the elderly demonstrate temporal stability over a limited timeframe, and exhibit distinctive phylum proportions and significant variability.^16^ More specifically, *Bifidobacterium*, which dominates the gut microbiota of infants, experiences a substantial decrease in relative abundance as age increases.^15^ Besides, the elderly show elevated levels of *Escherichia coli* and *Bacteroidetes*.^17^ Nevertheless, the generalizability of prior findings in microbiome-wide association studies has been limited by small sample sizes. In addition, studies have often focused on specific age groups, and neglected transitional periods and the whole lifespan. In addition, methodological variations, including DNA extraction, sequencing techniques, and bioinformatics analysis, hinder the comparison of results. These limitations impede the characterization of age-related features in the gut microbiota of women, and obscure our understanding of the associations between age and gut microbiota. Therefore, incorporating diverse populations across all stages of life with standardized methodologies becomes necessary.

Moreover, gaining insights into the effects of age on gut microbiota and its association with disease is important in enhancing our understanding of women’s health. Age-related alterations of gut microbiota not only contribute to dysregulated immune responses and immune system dysfunction, but also affect nutrient metabolism and the production of metabolites, such as short-chain fatty acids (SCFAs).^18,19^ Besides, age-related dysbiosis of gut microbiota can also disrupt the gut barrier function and lead to increased translocation of bacterial components into the bloodstream, thereby contributing to systemic inflammation.^20^ Even in the case of non-age-related diseases, such as inflammatory bowel disease (IBD), the phenotype and natural progression vary based on the age of onset.^21^ This suggests that age-related changes in gut microbiota might be involved in the pathophysiology of diseases. However, there is a dearth of studies comprehensively examining the associations between age-related changes in gut microbiota and diseases among women. Moreover, the influence of age on the disease-gut microbiota associations, along with the age-specific microbial features of the disease, remains largely unknown.

Therefore, our objective was to explore age-related features of gut microbiota throughout women’s lifespan, and determine their associations with women’s health. In addition, we aimed to uncover the age specificity of disease-gut microbiota associations. This study could inspire improvements in healthy aging, promote disease prediction, and bolster personalized healthcare for women.

## Results

### Impacts of host factors on gut microbiota of women

**Figure 1.**
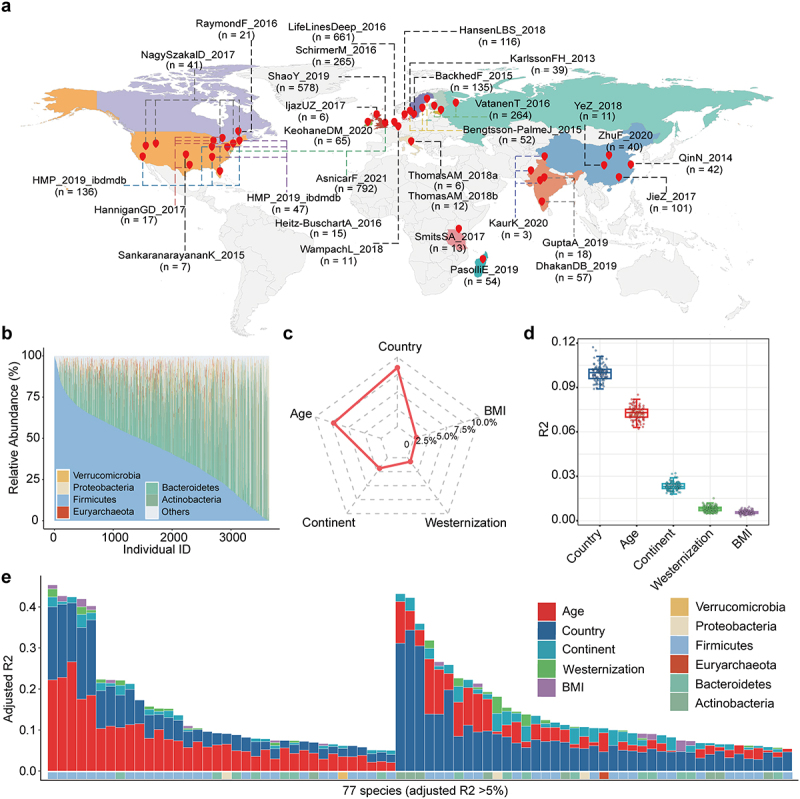
Outline of sample data and the influence of host factors on women’s gut microbiota. (a) Description of the sampling region (including 16 countries across 4 continents) and the metagenomic data size (*n* = 3625) used in the study. The detailed information of study name was given in Table S2. (b) the relative abundance of the top 6 gut microbiota in the level of phylum among subjects. The remaining microbiota were categorized as “others”. (c) amount of the variance (R^2^) in gut microbiota attributed to host factors, as assessed using PERMANOVA analysis (all *P* = .001). DNA extraction kit and sequencing platform were considered as covariates. (d) the box plots indicating the effect distribution of host factors (R^2^) on gut microbiota using bootstrapped PERMANOVA analysis (all mean *P* < .05). The analysis was accomplished by sampling 20% subsets without replacement for 100 iterations. (e) the bar plot displaying variations in each species explained by individual host factors, as estimated through the linear regression method. Finally, 77 species with cumulative adjusted R^2^ >5% (FDR_*F*-test_ < .05) were shown.

As shown in Figure 1a and Table S1, the Healthy Cohort comprised 3625 subjects from 16 countries across the four continents. Following the filtering criteria of prevalence < 10% and average relative abundance < 0.005%, 169 species were included in the subsequent analysis. Permutational multivariate analysis of variance (PERMANOVA) was employed to assess the impact of technological factors on batch effects in the multi-cohort microbiome datasets. The results indicated significant effects of DNA extraction kit (R^2^ = 0.145, *P* = .001), sequencing platform (R^2^ = 0.044, *P* = .001), and median read length (R^2^ = 0.010, *P* = .001) on the species-level profiles. Upon adjustment using the MMUPHin framework, the distribution of these factors became more evenly dispersed, which resulted in an obvious reduction of batch effects. The R^2^ values for DNA extraction kit, sequencing platform, and median read length after adjustment were 0.096, 0.015, and 0.001, respectively (all *P* = .001) (Figure S1). Considering the marginal effect of median read length, only the DNA extraction kit and sequencing platform were treated as covariates in the following analysis.

At the phylum level, *Firmicutes* constituted the majority (42.6%), followed by *Bacteroidetes* (29.0%), *Actinobacteria* (15.5%), and *Proteobacteria* (3.9%) (Figure 1b). PERMANOVA analysis was conducted to explore the influence of age and other host factors on species-level profiles among healthy women. Country exhibited the strongest interaction with gut microbiota (R^2^ = 0.085), followed by age (R^2^ = 0.072) and continent (R^2^ = 0.020) (Figure 1c). To address potential bias resulting from unbalanced population distribution, bootstrapped PERMANOVA was applied to introduce randomness. The results of bootstrapped PERMANOVA confirmed the robustness of the order of effects for host factors (Figure 1d). Furthermore, the variation explained by individual host factor for each species was assessed, and the dominant factor was identified if it accounted for the highest proportion of the variation. Age dominantly accounted for the inter-individual variations in 36 species, and country dominantly explained the variations in 41 other species (Figure 1e, Table S3). In conclusion, age was a significant factor contributing to variation in the gut microbiota.

### Gut microbiota of women showed age-dependent patterns

**Figure 2.**
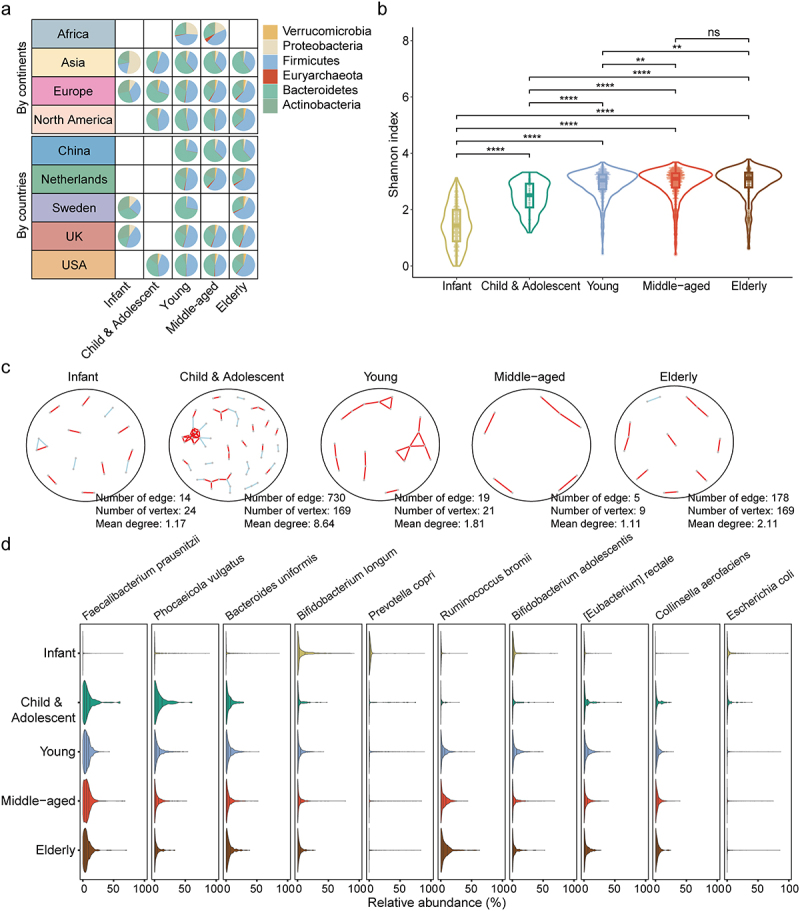
Identification of age-dependent pattern of gut microbiota among women. (a) temporal distributions of gut microbiota in the level of phylum across 4 continents and 5 countries. (b) the comparison of microbial alpha diversity across different age groups using Wilcoxon rank sum test. The Shannon index was estimated at the species level. ** .001 ≤ FDR < .01, *** .0001 ≤ FDR < .001, **** FDR < .0001. (c) co-occurrence network and network connection parameters across different age groups. In the network representation, each node corresponded to a distinct species, while the edges represented significant correlations between pairs of species (FDR < .05) with the absolute value of Spearman’s rho ≥ 0.4. Positive correlations were denoted by red edges, whereas negative correlations were represented by light blue edges. (d) the distribution of top 10 most abundant species across age groups.

To further investigate the dynamics of gut microbiota in healthy women across different age groups, temporal changes across four continents and five countries were initially examined. While regional variations had impacts on gut microbiota composition, distinct temporal changes were also observed within each continent and country. For example, in Asian infant women, *Proteobacteria* constituted more than 50% of the gut microbiota initially, but experienced a significant decline, giving way to the dominance of *Firmicutes* and *Bacteroidetes*. Consistent with findings from European populations, *Proteobacteria* exhibited enrichment during infancy, while *Firmicutes* exhibited a gradual increase throughout the lifespan of women in Sweden and the UK (Figure 2a).

Next, comparative analyses of microbial features were conducted, including diversity and co-occurrence networks, across different age groups. The Shannon index showed a significant increase from infants to middle-aged adults among healthy women (Wilcoxon rank sum test, all false discovery rate (FDR) < .01). However, the Shannon index reached a stable level in middle-aged and elderly adults (Figure 2b). In addition, the connectivity of species within the gut microbial network was highest among children and adolescents, as evidenced by the number of edges (730), vertices (169), and mean degree (8.64) (Figure 2c).

Moreover, the relative abundance of 169 species was compared among different age groups, and significant differences were observed in 167 species (Kruskal-Wallis rank sum test, all FDR < .01) (Table S4). In addition, age-dependent patterns of specific species were investigated by analyzing the temporal distributions of the top 10 most abundant species (Figure 2d). The majority of species exhibited an increasing pattern across the lifespan, whereas a few species showed a declining pattern. For example, *Faecalibacterium prausnitzii*, *Phocaeicola vulgatus*, and *Bacteroides uniformis* were found to be deficient during infancy, but experienced significant increases during childhood and adolescence, and remained stable in subsequent age groups. Conversely, *Bifidobacterium longum* displayed the highest abundance during infancy, but gradually decreased during the developmental process. We further examined the temporal distribution of these species in each continent and country, and revealed notable differences in their relative abundances across age (Figure S2). Interestingly, no prominent continent- or country-specific changes in species were observed, indicating that regional factors might not directly contribute to age-related variations in the gut microbiota.

### The aging process in women is characterized by age-specific species markers

**Figure 3.**
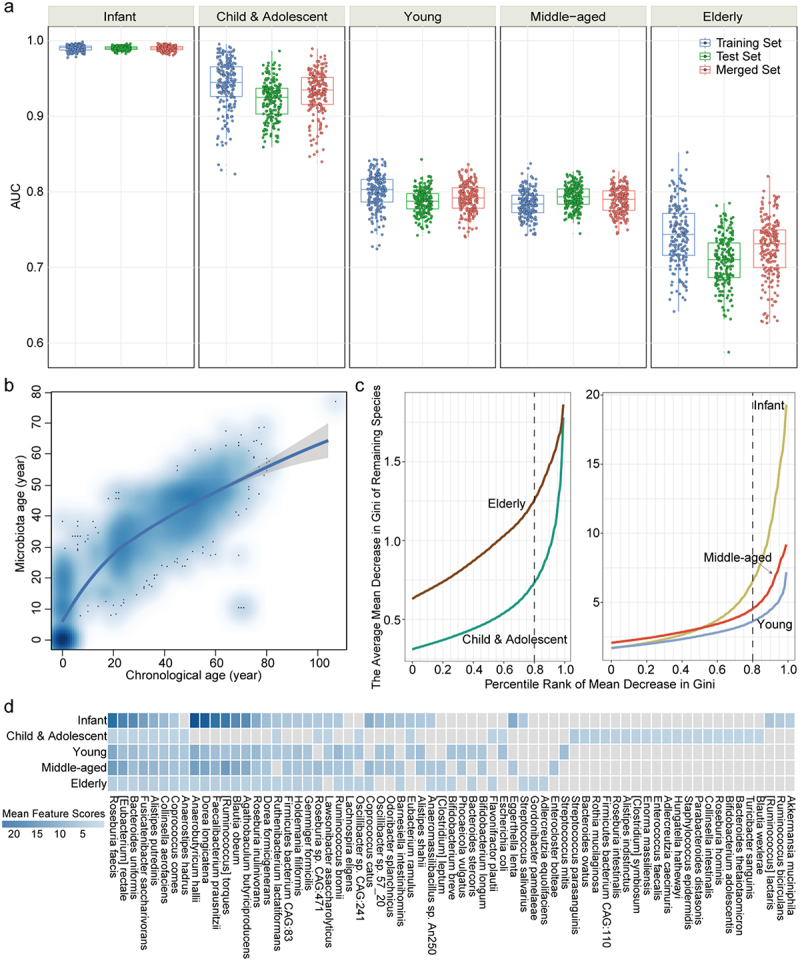
Age-specific features of gut microbiota among women. (a) the box plots illustrating the distribution of AUC values when the classifier was assessed on the training, test and merged sets across age groups. Each data point represented the median AUC from 10 repeated testing iterations, resulting in a total of 200 data points for each set. These calculations were performed by the ISSRF model. (b) the longitudinal progression of maturation of gut microbiota, as examined by assessing the microbiota age across the lifespan. The blue cloud symbolized the local density estimated from the spatial distribution of stool samples obtained from female subjects. (c) the fluctuations in mean feature scores of species across percentile ranks. The dotted black lines represented the percentile threshold used to identify age-specific species markers. (d) the heatmap showing the average mean decrease in Gini for age-specific species markers across diverse age groups. The cells in gray signified empty values, indicating that the corresponding species did not serve as an age-specific marker. Abbreviation: AUC: area under curve.

In light of the age-dependent pattern in the gut microbiota among women, further investigations were conducted to determine whether a certain list of species could differentiate between age groups. To minimize potential biases arising from outlier values and class imbalances, as well as the influence of other host factors, such as country and continent, the iterative sub-sampling based random forest (ISSRF) model was applied, which introduced additional randomness through sub-sampling. As depicted in Figure 3a, the results demonstrated moderate to good predictive accuracy, with area under the curve (AUC) values ranging from 0.709 ± 0.033 (elderly, test set) to 0.990 ± 0.002 (infant, test set). The AUC values obtained from the training, test, and merged sets were generally consistent, with only subtle differences observed between the groups, which confirmed the robustness of age-specific species markers. Notably, a declining trend in AUC values of gut microbiota from infancy to old age was observed, suggesting reduced discriminatory power of gut microbiota in predicting age as individuals aged. This phenomenon implied that the composition and functional characteristics of gut microbiota become less distinctive and more diverse with advancing age. In turn, we investigated the prediction performance of gut microbiota on chronological age by modeling the microbiota age. After adjusting for host factors and technological variations, increasing pattern of microbiota age against chronological age was observed across the entire lifespan and within each age group (Figure 3b Figure S3). The results suggested that the composition of gut microbiota undergoes progressive transitions as individuals age. Overall, the findings provided evidence that age can be directly associated with changes in gut microbiota, and the maturation of gut microbiota follows a consistently increasing pattern with chronological age.

In addition, the ISSRF model provided a list of species, along with their mean decrease in Gini values across 200 iterations, which allowed us to identify age-specific species markers. The percentile threshold for the normalized mean decrease in Gini was determined to identify the optimal set of markers. The mean feature scores for all age groups remained consistently low until the 80% threshold, after which they showed a significant increase (Figure 3c). Using an 80% threshold, 34 species markers for each age group were identified, resulting in a total of 67 markers. The average mean decrease in Gini of these 67 species markers was then compared across different age groups, and all markers showed significant differences among age groups (Kruskal-Wallis rank sum test, all FDR < .01), indicating their discriminatory power in distinguishing between age groups (Table S5). Interestingly, most age-specific species markers were shared among two or more age groups, while only a limited number of unique species markers were found in each group, ranging from one in young and middle-aged adulthood to 19 in childhood and adolescence (Figure 3d). This observation suggested that shared species may play essential roles in core functions of gut microbiota, but also undergo gradual changes as individuals age.

### Associations between disease and gut microbiota of women are impacted by age

**Figure 4.**
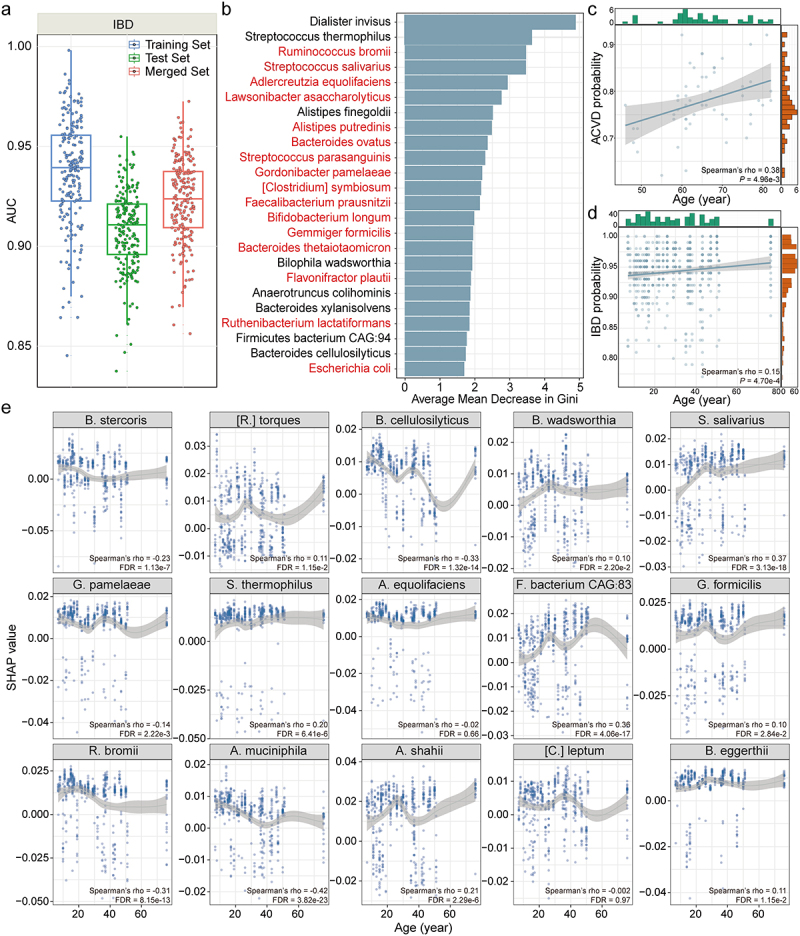
Impacts of age on the associations between disease and gut microbiota among women. (a) the box plots displaying the distribution of AUC values when the IBD classifier was examined on the training, test and merged sets. Each data point represented the median AUC from 10 repeated testing iterations, resulting in a total of 200 data points for each set. These calculations were performed by the ISSRF model. (b) the bar plot illustrating the average mean decrease in Gini for species markers associated with IBD. The species marked in red represented the identified age-specific markers of healthy women within corresponding age groups. (c) Spearman correlation between age and predicted ACVD probability estimated from SHAP. (d) Spearman correlation between age and predicted IBD probability estimated from SHAP. (e) Spearman correlations between age and SHAP values of 15 IBD species markers. FDR was calculated based on Benjamini-Hochberg procedure. Abbreviations: IBD: inflammatory bowel disease; ACVD: atherosclerotic cardiovascular disease.

Furthermore, we extended the focus to the role of age in disease-gut microbiota associations among women, as well as the relevance of identified age-specific species markers to these associations. Four diseases were selected within the Diseased Cohort: IBD, type 2 diabetes (T2D), atherosclerotic cardiovascular disease (ACVD), and impaired glucose tolerance (IGT) (Table S6). After identifying and addressing batch effects, age remained a significant contributor to the variation in gut microbiota, as indicated by both PERMANOVA and bootstrapped PERMANOVA analyses (Figure S4, S5). The ISSRF model revealed that IBD showed good and robust predictive accuracy, and the AUC values for the training, test, and merged sets were 0.937 ± 0.026, 0.908 ± 0.020, and 0.923 ± 0.021, respectively (Figure 4a). However, ACVD, IGT, and T2D exhibited unstable AUC values and evident overfitting, as higher AUC estimates in the training set than in the test set (Figure S6a). This discrepancy might be attributed to the limited sample size of disease (*n* = 45–53) and the inability of resampling techniques to adequately address this issue. Therefore, only the IBD species markers were identified using the ISSRF model. Of the 24 identified species markers, significant differences in relative abundance across the age groups of IBD subjects were observed in 20 (83.3%) species (Kruskal-Wallis rank sum test, all FDR < .05) (Table S7, Figure S6b). Remarkably, 16 (66.7%) markers were also age-specific species from the age groups of IBD subjects (Figure 4b). These findings implied that age may play a role in shaping gut microbiota and its association with disease.

Because local explanations at the individual level could enhance robustness against data variations and mitigate the risks associated with limited datasets, Shapley Additive Explanations (SHAP) was utilized. First, the SHAP value of each species and the base value were summed to calculate the predicted probability of disease. Age was positively correlated with the predicted probability of ACVD (Spearman’s rho = 0.38, 95%CI:0.11, 0.60; *P* < .01) and IBD (Spearman’s rho = 0.15, 95%CI:0.06, 0.23; *P* < .001) (Figure 4c , d). Second, the summary plot displayed the global feature importance of the top 15 species, of which 10 (66.7%), 5 (33.3%), 4 (26.7%), and 2 (13.3%) were age-specific species markers from the age groups of IBD, ACVD, IGT, and T2D, respectively (Figure S6c-f). Third, Spearman correlation analysis revealed that the SHAP values of 13 IBD species markers (86.7%) were significantly correlated with age (Spearman’s rho = −0.42 to 0.37; all FDR < .05) (Figure 4e). The findings reinforced the impact of age on disease-gut microbiota associations, and age-specific species markers might play a role in the pathogenesis and development of disease among women.

### Age specificity is identified in the associations between disease and gut microbiota of women

**Figure 5.**
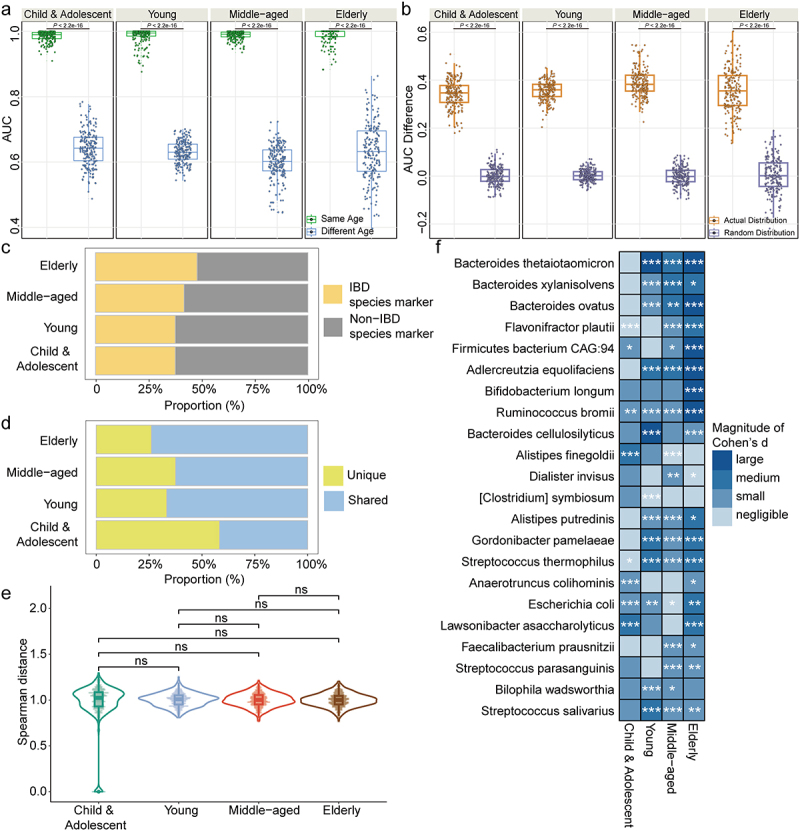
Identification of age specificity of the associations between disease and gut microbiota among women. (a) the box plots illustrating the distributions of AUC values when IBD classifiers trained on one age group were assessed on the same or different age groups. Each data point denoted the median AUC from 10 repeated testing iterations, resulting in a total of 200 data points for each set. The ISSRF model was utilized to perform these calculations. Comparisons between AUC values were performed using Wilcoxon signed rank test. (b) the box plots exhibiting the comparison of actual AUC differences obtained from IBD classifiers trained on one age group with the random distribution of AUC differences derived from two permuted sets. Each data point denoted the difference of median AUC from 10 repeated testing iterations, resulting in a total of 200 data points for each set. The ISSRF model was utilized to perform these calculations. Comparisons between AUC differences were performed using Wilcoxon signed rank test. (c) the proportion of IBD (derived from Figure 4b across the whole IBD subjects and corresponding healthy controls) and non-IBD species marker across age groups. (d) the proportion of unique (those appearing only once within a single age group) and shared (those appearing multiple times across age groups) IBD age-specific species marker across age groups. (e) the distribution of Spearman distances for IBD age-specific species markers across age groups. IBD species markers specific to childhood and adolescence were chosen. Subsequently, Spearman distances were calculated to determine the dissimilarity between the mean feature scores of these markers and the same markers both within the same age group and across other age groups. The comparison of Spearman distances was conducted using Wilcoxon rank sum test. ns: non-significant. (f) the heatmap presenting the magnitude of Cohen’s d of selected species markers across different age groups. These selected markers were IBD species markers from Figure 4b that additionally replicated in the age groups. * .01 ≤ FDR < .05, ** .001 ≤ FDR < .01, *** .0001 ≤ FDR < .001. Abbreviations: AUC: area under curve; IBD: inflammatory bowel disease.

Inspired by the impact of age on disease-gut microbiota associations, we further investigated the presence of age specificity. Regarding statistical power, only the age specificity of the associations between IBD and gut microbiota was explored using the ISSRF model. The classifier exhibited excellent predictive accuracy within the same age group, with AUC values ranging from 0.984 to 0.989. However, when tested on the different age groups, the predictive accuracy significantly decreased (Wilcoxon signed rank test, all *P* < 2.2e^−16^) (Figure 5a). To validate that the difference in median AUCs between the same and different age groups was not due to chance, age-group labels were permuted. The difference in median AUCs obtained from the actual distribution was significantly higher than that from the random distribution (Wilcoxon signed rank test, all *P* < 2.2e^−16^) (Figure 5b). Using an 85% threshold, 24, 24, 24, and 23 IBD age-specific species markers were identified for childhood and adolescence, young adulthood, middle-aged adulthood, and old age, respectively (Figure S7a, b). In addition, the impact of age on gut microbiota accounted for higher variation (R^2^ = 0.047) compared to the variation explained by health status in the IBD sub-cohort (R^2^ = 0.009) (Figure S8). This observation reinforced the influence of age specificity on the association between IBD and gut microbiota.

Next, the proportion of IBD species markers exhibited an upward trend across age groups, increasing from 37.5% (9/24) in childhood and adolescence to 47.8% (11/23) in the elderly (Figure 5c). However, the proportion of unique species markers displayed a reduced trend across age groups, decreasing from 58.3% (14/24) in childhood and adolescence to 26.1% (6/23) in old age (Figure 5d). This difference was statistically significant between childhood/adolescence and old age (chi-square test, *P* = .025). These findings suggested the loss of IBD-specific microbial features and increased microbial variability during childhood and adolescence.

To validate these findings, the stability of IBD age-specific species markers was examined. We selected markers specific to childhood and adolescence, and calculated Spearman distances to assess the dissimilarity between their mean feature scores within the same age group and across other age groups. No significant differences were observed in childhood and adolescence compared with young and middle-aged adulthood and old age (Figure 5e). Moreover, Spearman distances within the same age group, including young and middle-aged adulthood and the elderly, were consistently lower than those across different age groups (Wilcoxon rank sum test, all FDR < .05) (Figure S7c-e). These findings suggested that IBD age-specific species markers in childhood and adolescence may exhibit reduced reproducibility and stability, whereas age-specific species markers in other age groups remain consistently stable.

To shed light on the underlying reasons for this instability, we examined the IBD species markers that was replicated across age groups, and compared the effect size of these species between IBD subjects and healthy controls. Childhood and adolescence were characterized by a decrease in the number of significantly different effect size and a reduction in the magnitude of the effect size, which could contribute to the masking of IBD species markers (Figure 5f). Therefore, age specificity may reduce the susceptibility of children and adolescents to IBD species markers.

## Discussion

Based on multi-cohort microbiome studies, we provided the landscapes of age-dependent features of gut microbiota, and determined age-specific species markers throughout the lifespan of women. Additionally, age could influence disease-gut microbiota associations, and age-specific species markers might play a role in the pathogenesis of diseases. Finally, the age specificity could impact the stability and reproducibility of disease species markers. For instance, age specificity may lower the susceptibility of children and adolescents to IBD species markers.

The strong association between country and gut microbiota in our study underscored the significant impact of geographic location on microbial composition. It is well established that region-related environmental conditions, such as climate fluctuations, greenspace availability, urbanization, and pollution levels, are involved in shaping the human gut microbiota.^22^ Besides, prior investigations have consistently revealed disparities in gut microbiota across diverse regions, which were driven by variations in dietary patterns, lifestyle factors, and cultural norms.^23,24^ To mitigate the confounding effects of geographical factors, several steps were implemented. First, the ISSRF model was employed, which involved subsampling and conducting multiple iterations to test the classifier. The median AUC value was selected as a representative measure. Second, when modeling the microbiota age, country, continent and other host factors were included as covariates, and the resulting residuals were used for analysis. Third, only healthy controls from the same age group and country as the diseased population were included. These procedures aimed to decouple the influence of geographical factors and provide a more accurate assessment of the role of age.

A distinct transition in the predominant phyla of gut microbiota was evident among women at various life stages. In line with prior studies, *Proteobacteria* dominates gut microbiota during infancy, which is potentially shaped by factors such as breast milk composition, delivery mode, and exposure to environmental microbes.^25,26^ In addition, the shift from *Proteobacteria* to *Firmicutes* and *Bacteroidetes* coincides with the introduction of solid foods.^27^ The enrichment of *Firmicutes* and *Bacteroidetes* in the gut microbiota of adult women corresponds to their involvement in carbohydrate metabolism and the production of SCFAs.^28^ In addition, the Shannon index exhibited a noteworthy rise from infancy to middle-aged adulthood, indicating the progressive diversification of gut microbiota. These results were in line with previous research emphasizing the dynamic nature of gut microbiota in early life and its gradual maturation in response to dietary habits, lifestyle factors, and environmental exposures.^15^ Besides, childhood and adolescence represent the formation of complex microbial communities characterized by heightened connectivity. The presence of dense networks facilitates metabolic cross-feeding, which, in turn, enhances nutrient utilization, strengthens ecosystem resilience, and contributes to the stability of the microbial ecosystem.^29^ Furthermore, increment in abundance was observed among most species, signifying the gradual microbial colonization throughout the lifespan. The early enrichment of *Bifidobacterium* species can be attributed to their ability to metabolize breast milk oligosaccharides.^30^ Nevertheless, as children transition to solid foods and advance in age, the relative abundance of *Bifidobacterium* declines.

This study revealed a declining trend in the AUC values of gut microbiota from infancy to old age. During the transition from infancy to childhood and adolescence, gut microbiota undergo dynamic changes influenced by factors such as diet, hormonal fluctuations, and immune system maturation.^15,31,32^ Age-related physiological changes, including reduced immune function, alterations in gut motility, and changes in nutrient absorption, also affect the composition and diversity of gut microbiota.^33^ Consequently, gut microbiota becomes more distinct and individualized with increasing age. Shared age-specific species markers implied the presence of core microbial taxa that sustain their functions throughout the lifespan, aligning with prior research emphasizing the role of core microbiota despite age-related variations.^34^ For example, the shared *Bacteroides uniformis* has been associated with host health through the production of SCFAs, involvement in metabolism and weight regulation, and its immunomodulatory properties.^35^ In addition, unique species markers in each age group highlighted the specific microbial features within certain life stages. For example, *Bifidobacterium adolescentis*, *Bacteroides thetaiotaomicron*, and *Bacteroides ovatus* were age-specific species markers in women during childhood and adolescence, possibly influenced by the transition to solid foods. *Bacteroides* species can degrade complex carbohydrates and ferment dietary fibers, which aligns with the dietary diversification during the periods.^36^ *Bacteroides* species are also crucial for immune training and maturation during childhood and adolescence.^35^ Moreover, several age-specific species markers have also been reported to be disease-related. For instance, *Collinsella aerofaciens* is related to colorectal cancer,^37^ *Dorea longicatena* to obesity,^38^ and *[Ruminococcus] torques* to diabetes,^39^ suggesting the broad implications of age-specific species for diseases. Our findings could have implications for gut microbial assessments across the lifespan of women. Based on early identification of imbalances, dietary plans and probiotic regimens might be conducted to address the unique microbial needs of women in different life stages, promoting gut health and overall well-being.

Several disease species markers in our study have not been previously documented in the original literature.^40,41^ This inconsistency could be attributed to disparities in the methodologies, and the imbalances in sample distribution across age groups from the original studies. In addition, we exclusively included female subjects, and integrated multiple studies to ensure statistical power. Our findings underscored the effects of age and age-specific species markers on the associations between diseases and gut microbiota. For instance, *Ruminococcus bromii* was an age-specific marker among the elderly, consistent with previous research indicating positive associations between *Ruminococcus* species and advancing age.^42^ *Ruminococcus* species have been reported as producers of SCFAs and major amino acid-fermenting bacteria, whose decrease in abundance was associated with intestinal inflammation.^43^ The study also indicated that *Ruminococcus bromii* was a species marker for IBD, and decrease in its abundance might elevate the risk of IBD. Moreover, consistent with prior research, the age-related patterns of *Streptococcus salivarius* and *Streptococcus parasanguinis* were observed.^42^ Multiple *Streptococcus* members have been extensively investigated because of their capacity to generate toxins, adhesins, and hydrogen peroxide,^44,45^ resulting in heightened pro-inflammatory responses and increased risk of ACVD.^46,47^ Our results implied that integrating age-specific species markers into clinical practice could ensure more accurate diagnostics and treatment planning of women. Besides, health education for women about impacts of age on gut microbiota and its relevance with disease, could empower them to make informed lifestyle choices that support gut health at different life stages.

Furthermore, age-specific disease markers suggested the importance of considering age in disease prediction, and possibly, the gut microbiota-targeted therapy. Taking IBD as an example, due to the reduced stability of IBD species markers among children and adolescents, the population might be less susceptible to IBD species markers. Since dysbiosis of gut microbiota was involved in the pathogenesis of IBD, our finding might explain the lowest incidence rate of IBD among children and adolescents.^48,49^ Moreover, the reduced differences of effect size in childhood and adolescence might be attributed to the dense microbial network mentioned in the contents of age-dependent patterns, which enhanced the ecosystem’s resilience against IBD-related pathogens. However, the underlying mechanisms of age-specificity in IBD-gut microbiota associations warrant further investigation.

Our study had several strengths. First, landscapes of age-dependent microbial features and age-specific species markers were characterized, shedding light on the role of age in the gut microbiota. Second, we firstly determined the impact of age, as well as age-specific species markers, on the associations between disease and gut microbiota in women. Third, several state-of-the-art analysis techniques, such as bootstrapped PERMANOVA and the ISSRF model, were employed to address the challenges commonly encountered in multi-cohort microbiome studies, such as outliers, correlated errors, and class imbalance. However, this study had several limitations. First, owing to the unavailability of metadata, other host factors such as diet, medication intake, and disease subtypes were not studied. Future population-based studies that extensively phenotype individuals are needed to elucidate the complex relationships between gut microbiota and host factors. Second, our analyses were based on cross-sectional observational data. Caution should be made when interpreting these associations, and longitudinal follow-up and intervention studies are necessary to establish causality. Third, the participants in the Diseased Cohort were limited to specific countries, which might impede the generalizability of our findings. Access to emerging microbiome datasets from different regions is crucial for cross-cohort validations.

In summary, we comprehensively described temporal dynamics of gut microbiota, and determined age-specific species markers of women. Moreover, our study provided in-depth insights into the effects of age and age-specific species markers on disease-gut microbiota associations among women. These findings could inspire for improving healthy aging, enhancing disease prediction, and supporting personalized healthcare for women.

## Materials and methods

### Sample collection

The study utilized data from the curatedMetagenomicData package (version 3.4.2), which is known for standardized and curated human microbiome data.^50^ Stool samples were selected from women (*n* = 8014), and 3426 subjects with current antibiotic use or unknown information about antibiotic usage were excluded from the analysis. Recent research has increasingly emphasized the alterations in gut microbial composition, abundance, and diversity during pregnancy,^51^ therefore 15 pregnant women were additionally excluded. Subjects labeled as “healthy” in the disease metadata category were included and grouped into the Healthy Cohort (*n* = 3629). Moreover, we excluded any single country or continent with fewer than 10 samples, resulting in a total of 3625 samples. Relevant metadata, including country, age, body mass index (BMI), and westernization, were retained to explore the influence of host factors on gut microbiota. Continent information was included as region-related metadata to comprehensively examine the potential impacts of regional factors. In the following analysis, microbial species with a prevalence ≥ 10% and an average relative abundance ≥ 0.005% remained.^52^

### Batch effect identification and calibration

Despite the standardized bioinformatic analysis approach employed for data from the curatedMetagenomicData package, potential biases could arise from the choice of sequencing technologies and sample preparation methods in multi-cohort microbiome datasets. These factors included the sequencing platform, median read length, and DNA extraction kit. To assess the impacts of these batch effects, Bray-Curtis dissimilarity matrix was constructed based on species-level compositional data. We then conducted permutational multivariate analysis of variance (PERMANOVA) using the adonis function from the vegan package (version 2.6.4) with 999 permutations to evaluate the contributions of these factors. In addition, classical metric multidimensional scaling was used to extract and visualize different principal coordinates based on the Bray-Curtis distance matrix.

Because of the zero-inflated and compositional characteristics of microbial abundance data, conventional batch correction methods developed for gene expression data, such as ComBat, were not directly applicable.^53^ Therefore, we adopted the zero-inflated empirical Bayes adjustment within the MMUPHin framework to adjust the effects of sequencing technologies and sample preparation methods.^54^ The batch effects resulting from these factors after adjustment were evaluated using the same methodology, which suggested an evident reduction.

### Impacts of host factors on gut microbiota

To assess the contribution of country, age, BMI, westernization, and continent to the variance in gut microbiota, PERMANOVA analysis was conducted with 999 permutations, with sequencing platform and DNA extraction kit adjusted. Given the unbalanced distribution of the population, bootstrapped PERMANOVA approach was employed to evaluate the robustness and stability of the PERMANOVA analysis. It involved randomly sampling 20% subsets from the entire population without replacement for 100 iterations, performing PERMANOVA on each sampled dataset, and summarizing the results across iterations.

### Estimating the variance of individual species

To evaluate the impacts of country, age, BMI, westernization, and continent on the variance of each species, a linear model was employed. By incorporating the centered log-ratio (CLR) transformed species as the dependent variable in the model, the variance associated with the aforementioned factors was estimated, and the adjusted R^2^ and *F*-test *P* values were documented. To address multiple comparisons, the false discovery rate (FDR) was calculated using the Benjamini-Hochberg procedure.

### Microbial feature comparisons across age and region groups

Age groups were defined as follows: infant (birth-1 year, *n* = 947), child (2–12 years, *n* = 96), adolescent (13–17 years, *n* = 81), young adult (18–39 years, *n* = 959), middle-aged adult (40–59 years, *n* = 1168), and elderly (≥60 years, *n* = 356). Owing to the limited sample size, the child and adolescent groups were combined. Only countries with a sample size > 5% of the Healthy Cohort population were included in the analysis, resulting in China (*n* = 194), the Netherlands (*n* = 926), Sweden (*n* = 226), the UK (*n* = 1311), and the USA (*n* = 311) being retained for inter-country comparisons. Comparisons were also performed across different continents, including Africa (*n* = 67), Asia (*n* = 272), Europe (*n* = 2952), and North America (*n* = 334).

First, the relative abundance of species among different age groups was compared using the Kruskal-Wallis rank sum test, and the Benjamini-Hochberg FDR was calculated for multiple testing adjustments. Second, the Shannon index was estimated using the diversity function from the vegan package (version 2.6.4), and comparisons across groups were performed using the Wilcoxon rank sum test with Benjamini-Hochberg FDR adjustment. Third, the network properties of microbial co-occurrence, including edge number, vertex number, and average degree, were assessed using the igraph package (version 1.4.2).

### Identifying age-specific species marker

To avoid potential bias from multi-cohort microbiome studies, including outlier values and class imbalance, the iterative sub-sampling based random forest (ISSRF) model was applied. In the first step, the species was CLR-transformed and one age group was chosen as the outcome, while the remaining groups were merged as the control. The Healthy Cohort was then randomly partitioned into a 50% training set and a 50% test set. The sample size for each iteration was fixed at 60% of the minimum number of outcomes in the training and test sets. In the second step, a classifier was constructed using the training set through random sampling with a fixed sample size. To ensure that variations in the prediction performance of age groups were not influenced by biased subsamples and overfitting, the classifier was tested on both the training set (excluding those selected during training) and the test set with a fixed sample size. The testing process was repeated 10 times, and the median area under the curve (AUC) was calculated. The above iterative sub-sampling based classifier was constructed for 200 iterations. In the third step, to mitigate the impacts of correlated errors and outlier values on prediction performance, the training set (excluding those selected during training) and the test set were merged. The outcome and control labels of the subjects were permuted and a permuted test set was created. For each of the 200 sub-sampling based classifier iterations, the classifier was evaluated 10 times on the permuted test set, and the median AUC was computed. Consequently, the iterative sub-sampling strategy employed in the ISSRF model introduced additional randomness by sub-sampling both observations and features. This approach effectively reduced overfitting, enhanced the generalization capability of the model, and ultimately improved the overall predictive performance.

To identify age-specific species markers, we determined the percentile threshold for the mean decrease in Gini. This involved calculating the average mean decrease in Gini for each species across 200 iterations and normalizing the values to a range of 0 to 1. By varying the threshold from 0 to 0.99, the arithmetic mean of the average mean decrease in Gini for species above the threshold was computed. Subsequently, we plotted the above arithmetic mean for the remaining species against their percentile ranks. The percentile threshold was selected as the inflection point where the mean feature scores showed a significant increase.

### Modelling microbiota age

In the Healthy Cohort, a linear regression model was applied, with the CLR-transformed relative abundance of species as the dependent variable. Covariates, such as country, continent, westernization, sequencing platform, and DNA extraction kit, were incorporated into the model to adjust for potential confounding factors. The residuals from the regression analysis were then used for subsequent analysis. To determine the minimum number of top-ranked age-discriminatory species required for prediction, the rfcv function from the randomForest package (version 4.7–1.1) was employed. This cross-validation prediction performance was conducted on the residuals for 100 iterations. Next, random forest (RF) regression was performed using the identified top-ranked age-discriminatory species, with the parameters set as ntree = 10000, nPerm = 100, and mtry = 1/3 of the minimum number of top-ranked age-discriminatory species.^55^ The predicted age from this model was considered as the microbiota age. Finally, a smoothing spline or linear function was fitted to the relationship between microbiota age and chronological age.

### Exploring the impacts of age on disease-gut microbiota associations

The selection criteria for women with specific diseases from curatedMetagenomicData package (version 3.4.2) were as follows: 1) stool samples; 2) without current antibiotic use; 3) complete values for age, country, and continent; 4) proportion of disease > 5% in the diseased population; and 5) availability of more than 10 samples from one country or continent. Consequently, individuals with inflammatory bowel disease (IBD; *n* = 537), type 2 diabetes (T2D; *n* = 45), atherosclerotic cardiovascular disease (ACVD; *n* = 53), and impaired glucose tolerance (IGT; *n* = 46) were included in the Diseased Cohort. To examine the association between gut microbiota and disease in women, efforts were made to match the diseased population with healthy controls. The selection of healthy controls took into account the effects of region and age on gut microbiota, and thus ensured that controls belonged to the same age group and region as the diseased population. Considering the finer resolution provided by country-level data and the greater contribution of country to gut microbiota variance compared to continent, we selectively included controls from the same country as the diseased population. Consequently, a total of 491 healthy controls from Healthy Cohort were included in the Diseased Cohort (*n* = 1172). Batch effects of gut microbiota were identified and adjusted using the aforementioned methodology. Additionally, the contribution of country, age, BMI, westernization, and continent to the variance in gut microbiota within the Diseased Cohort was evaluated using PERMANOVA and bootstrapped PERMANOVA analyses.

Then, the ISSRF model was applied to identify disease-specific species markers. Each disease, along with its matched controls, was randomly divided into a 50% training set and a 50% test set. The sample size for each iteration was fixed at 60% of the minimum number of disease in the training and test sets. A total of 200 iterations of the iterative sub-sampling based classifier were constructed on the CLR-transformed species, with the testing process repeated 10 times for each iteration.

Next, for each disease and its matched controls, we selected one age group as the training set, and combined the remaining age groups as the test set. The sample size for each iteration was fixed at 60% of the minimum number of diseases in the training and test set. The iterative sub-sampling based classifier was constructed on the selected age group for 200 iterations, and the testing process was repeated 10 times for both the same age group and different age groups. To determine if the difference in median AUC between the same and different age groups was not due to random chance, the age-group labels were permuted. The difference in median AUCs obtained from the actual test (same age group vs. different age group) and the permuted tests (permuted test set 1 vs. permuted test set 2) for the 200 iterations were compared using Wilcoxon signed rank tests.

### Identifying disease-gut microbiota associations based on local explanation

The Python library SHAP-based Microbiome Analyses Tool (SHAPMAT) (version 0.1.5), incorporating the local explanation technique called Shapley Additive Explanations (SHAP), was utilized for personalized feature importance identification.^56^ In this approach, SHAP values representing the contribution of each species to disease prediction were computed for each subject. By summing these SHAP values and the base value, the disease probability for each subject was determined. Spearman correlation analysis was then employed to assess the correlations between age and species-based disease probability. Subsequently, the top 15 species were selected as disease markers based on their mean absolute SHAP values, which indicated global feature importance. The correlations between age and SHAP values of these disease species markers were also evaluated using Spearman correlation analysis.

### Determining the stability of IBD age-specific species

The mean feature scores of the IBD age-specific species markers for each age group were selected. Spearman distances were used to measure the dissimilarity between the mean feature scores of selected species within the same age group and across different age groups. The Wilcoxon rank sum test was employed to compare Spearman distances among age groups, and the Benjamini-Hochberg FDR was applied for multiple testing adjustment. Lower Spearman distances indicated higher stability and reproducibility of age-specific species markers.

To unveil the reasons underlying the loss of stability and reproducibility, we examined IBD species markers in the IBD sub-cohort (consisting of both IBD subjects and corresponding healthy controls) that was replicated across age groups. The effect sizes of these species between IBD subjects and healthy controls were compared using Cohen’s d test. The Benjamini-Hochberg FDR was used for multiple testing adjustment. The magnitude of effect sizes was assessed using predefined thresholds: |d| < 0.2 was considered “negligible,” 0.2 ≤ |d| < 0.5 was categorized as “small,” 0.5 ≤ |d| < 0.8 was classified as “medium,” and otherwise denoted “large”.^57^

## Supplementary Material

Supplemental Material

## Data Availability

The processed data and codes supporting the findings of this study are available in the GitHub repository at https://github.com/TerenceDong/GutMircobiome_WomenHealth.
