# Supplementary material for: Disentangling the age-related manner in the associations between gut microbiome and women’s health: a multi-cohort microbiome study
Source: Gut Microbes. 2023 Dec 7;15(2):2290320. doi: 10.1080/19490976.2023.2290320 (PMC10730178; doi:10.1080/19490976.2023.2290320)
Supplement: Supplemental Material [file KGMI_A_2290320_SM1963.zip › Supplementary Figures (2).docx]

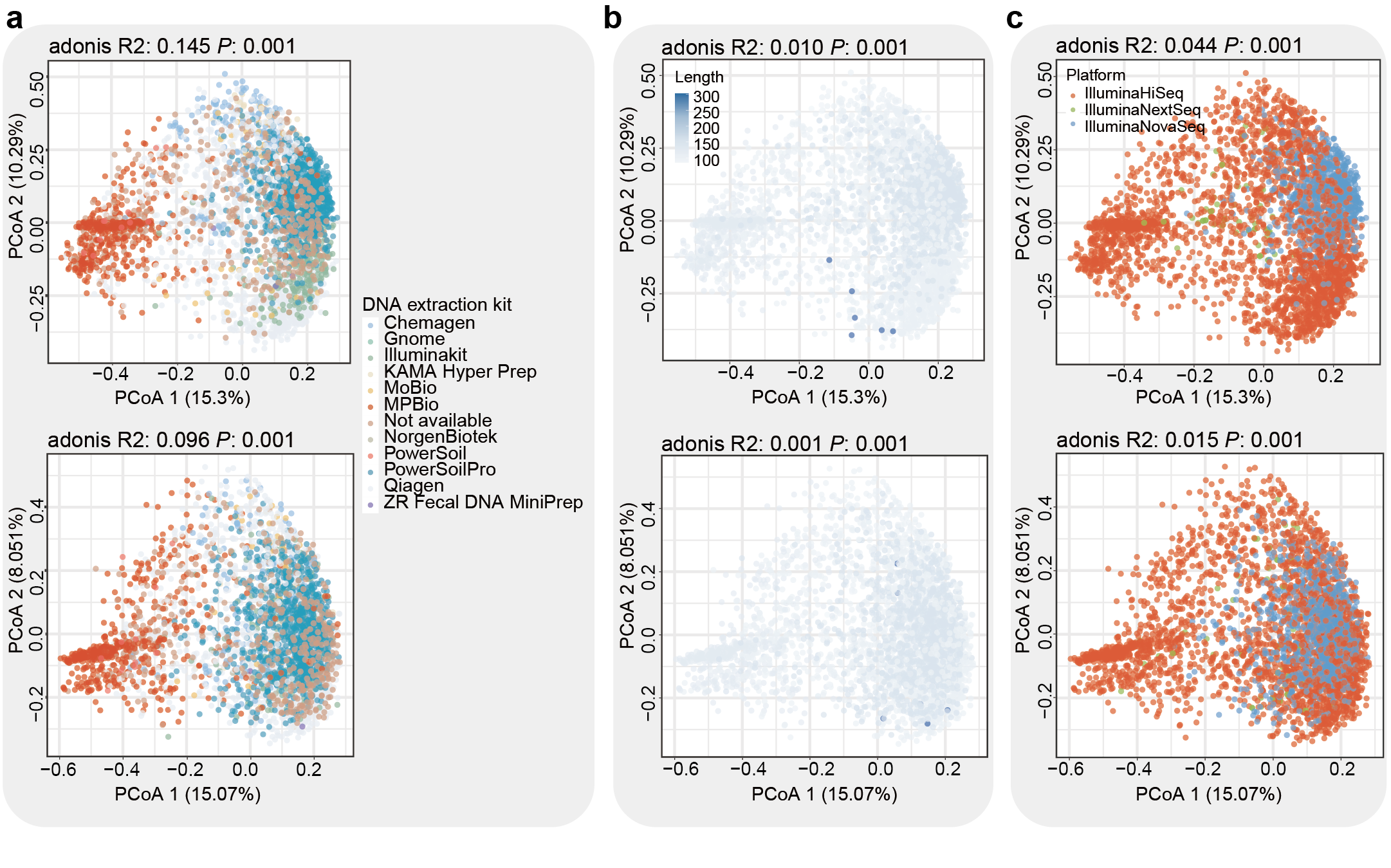
**Figure S1.** The amount of variance (R^2^) in women’s gut microbiota explained by technological factors before and after batch effects calibration. PERMANOVA analysis was conducted to quantify the impacts of (a) DNA extraction kit, (b) median read length and (c) sequencing platform on the structure of gut microbiota.


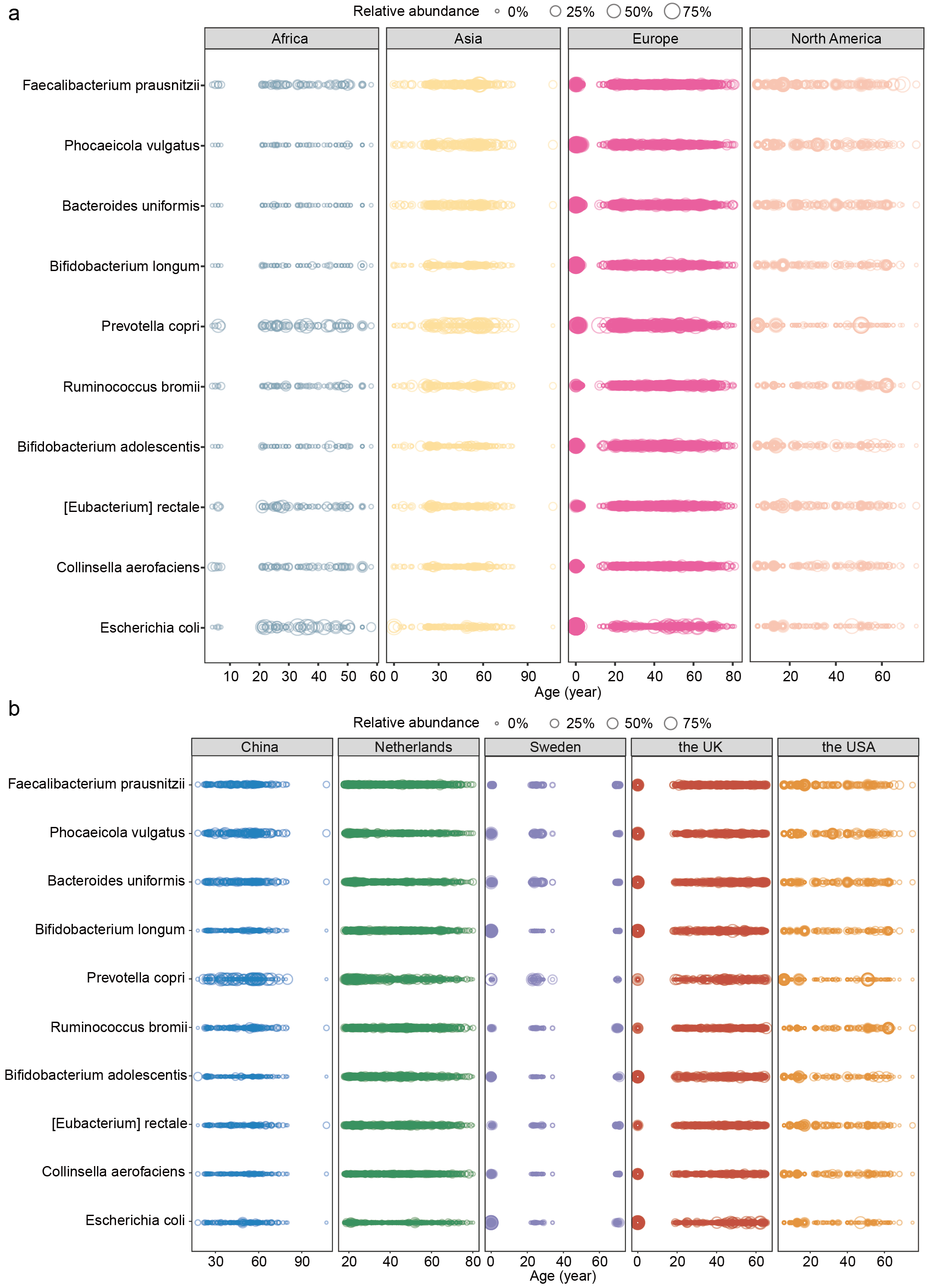


**Figure S2.** Temporal distribution of the top 10 species in the relative abundance among women subjects across different continents (a) and countries (b).


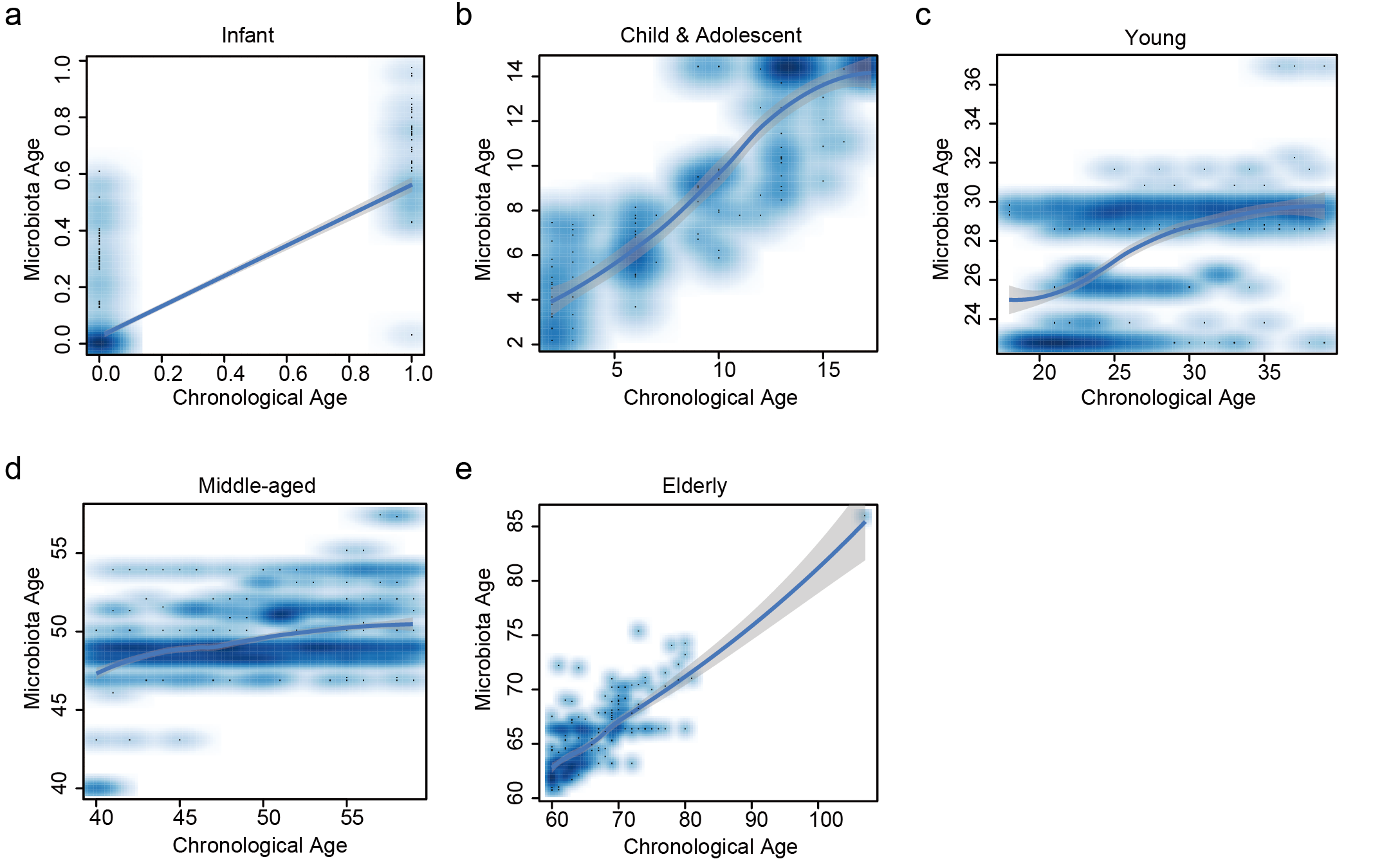


**Figure S3.** The longitudinal development of maturation of women’s gut microbiota. Microbiota age was evaluated across distinct age groups including (a) infants, (b) children and adolescents, (c) young adults, (d) middle-aged adults, and (e) elderly individuals. The blue cloud depicted the localized density estimation derived from the spatial distribution of stool samples.


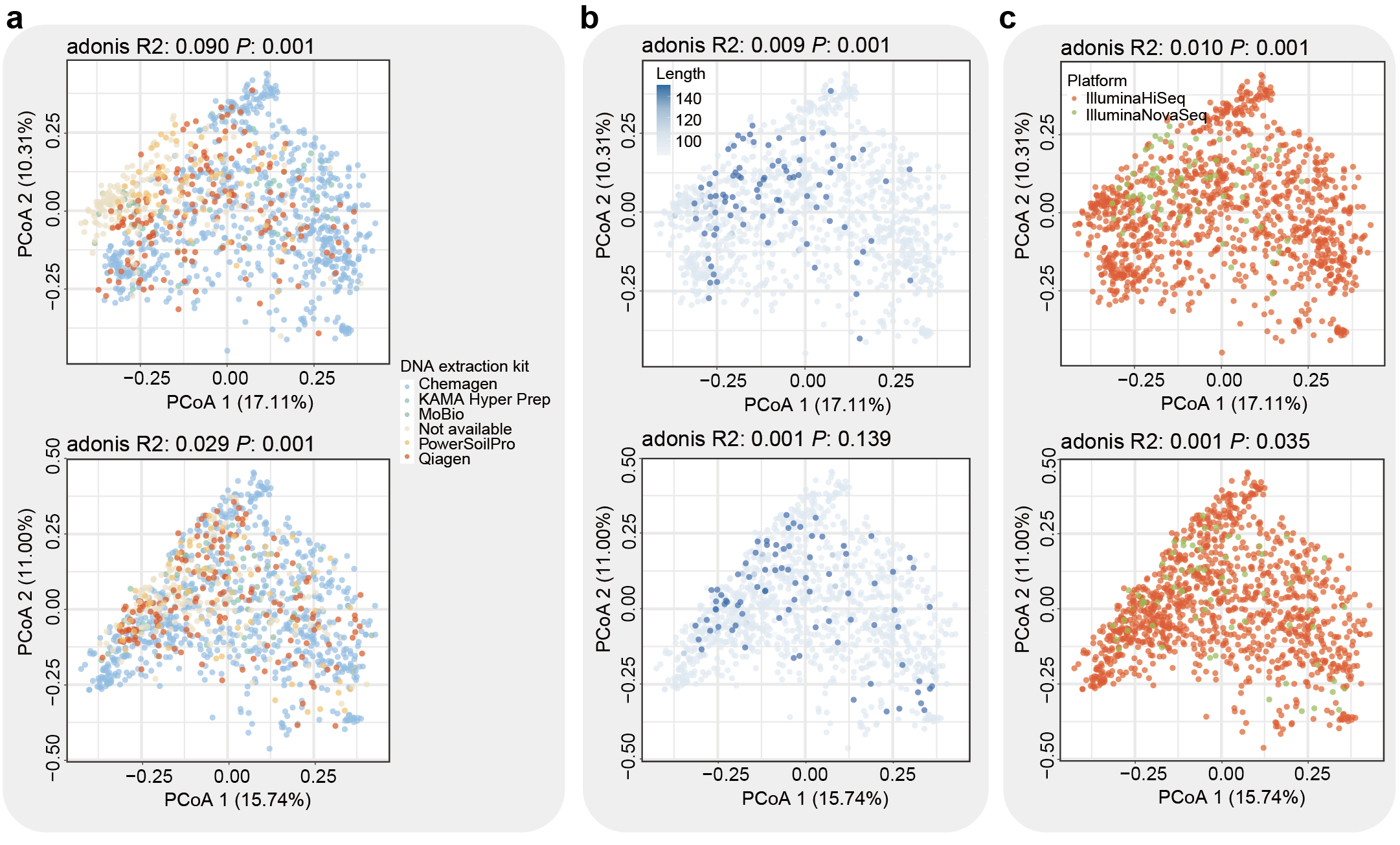


**Figure S4.** The amount of variance (R^2^) in the gut microbiota of the Diseased Cohort explained by technological factors before and after batch effects calibration. PERMANOVA analysis was conducted to quantify the impacts of (a) DNA extraction kit, (b) median read length and (c) sequencing platform on the structure of gut microbiota.


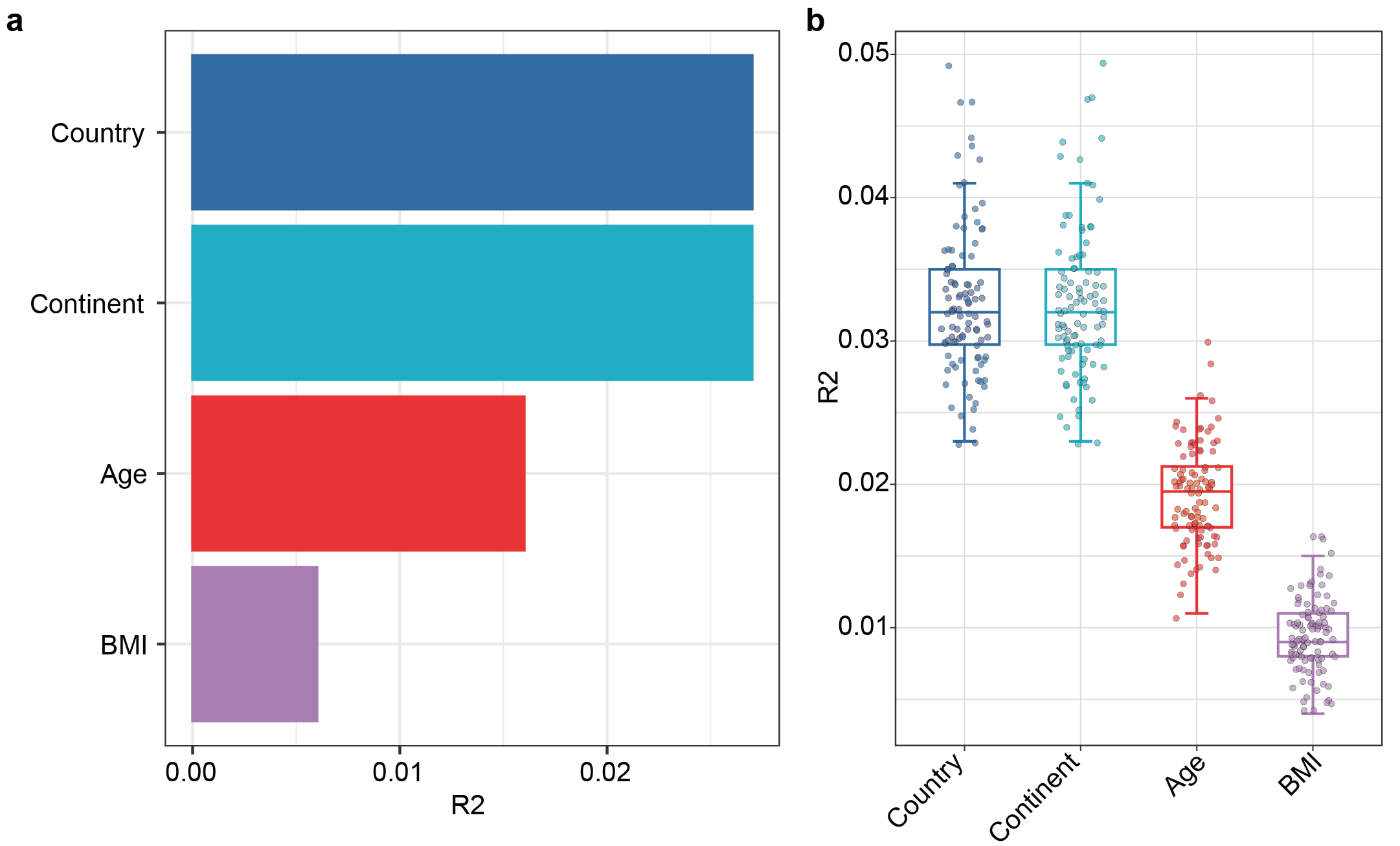


**Figure S5.** The impacts of host factors on gut microbiota of the Diseased Cohort. (a) Amount of the variance (R^2^) in gut microbiota attributed to host factors, as assessed using PERMANOVA analysis (all *P* = .001). DNA extraction kit was considered as covariates. (b) The box plots indicating the effect distribution of host factors (R^2^) on gut microbiota using bootstrapped PERMANOVA analysis (all mean *P* < .05).


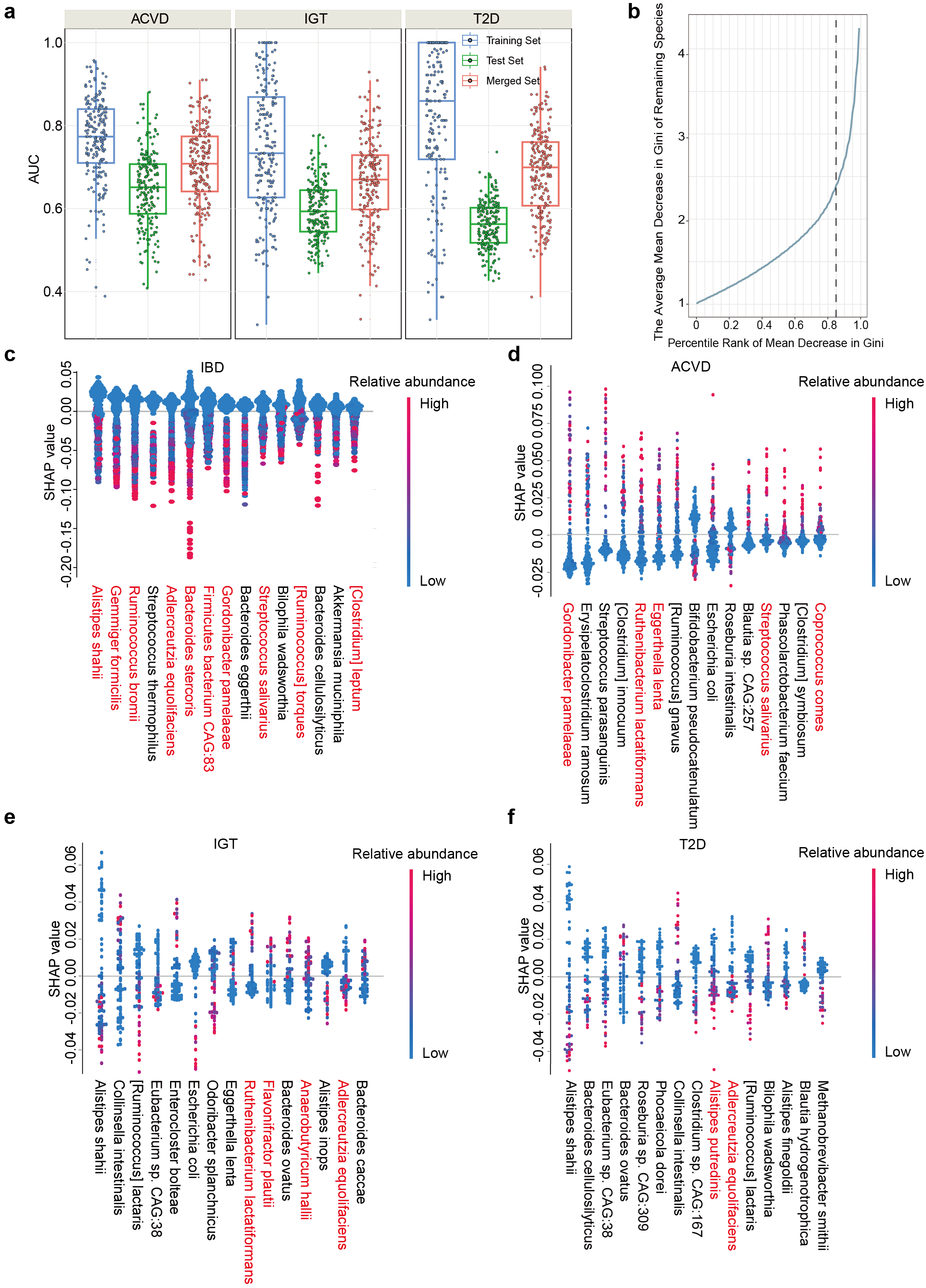


**Figure S6.** Identification of disease species markers among women. (a) The box plots displaying the distribution of AUC values when the ACVD, IGT and T2D classifier was examined on the training, test and merged sets. Each data point represented the median AUC from 10 repeated testing iterations, resulting in a total of 200 data points for each set. These calculations were performed by the ISSRF model. (b) The fluctuations in mean feature scores of species across percentile ranks. The dotted black lines represented the percentile threshold used to identify IBD species markers. (c) The summary plot showing the global feature importance of the IBD species markers. The species marked in red represented the identified age-specific markers of healthy women within corresponding age groups. The summary plot provided a visual representation of the SHAP values for all subjects, with each point signifying a subject's value. The species were arranged in descending order based on their mean absolute SHAP values. The position of the point on the y-axis reflected the influence of a particular species on the classifier's prediction for a given individual. Besides, the color of the point corresponded to the relative abundance of the species, where blue indicated low abundance and red indicated high abundance. (d) The summary plot showing the global feature importance of the ACVD species markers. The species marked in red represented the identified age-specific markers of healthy women within corresponding age groups. (e) The summary plot showing the global feature importance of the IGT species markers. The species marked in red represented the identified age-specific markers of healthy women within corresponding age groups. (f) The summary plot showing the global feature importance of the T2D species markers. The species marked in red represented the identified age-specific markers of healthy women within corresponding age groups. Abbreviations: AUC: area under curve; IBD: inflammatory bowel disease; ACVD: atherosclerotic cardiovascular disease; IGT: impaired glucose tolerance; T2D: type 2 diabetes; SHAP: Shapley Additive Explanations.


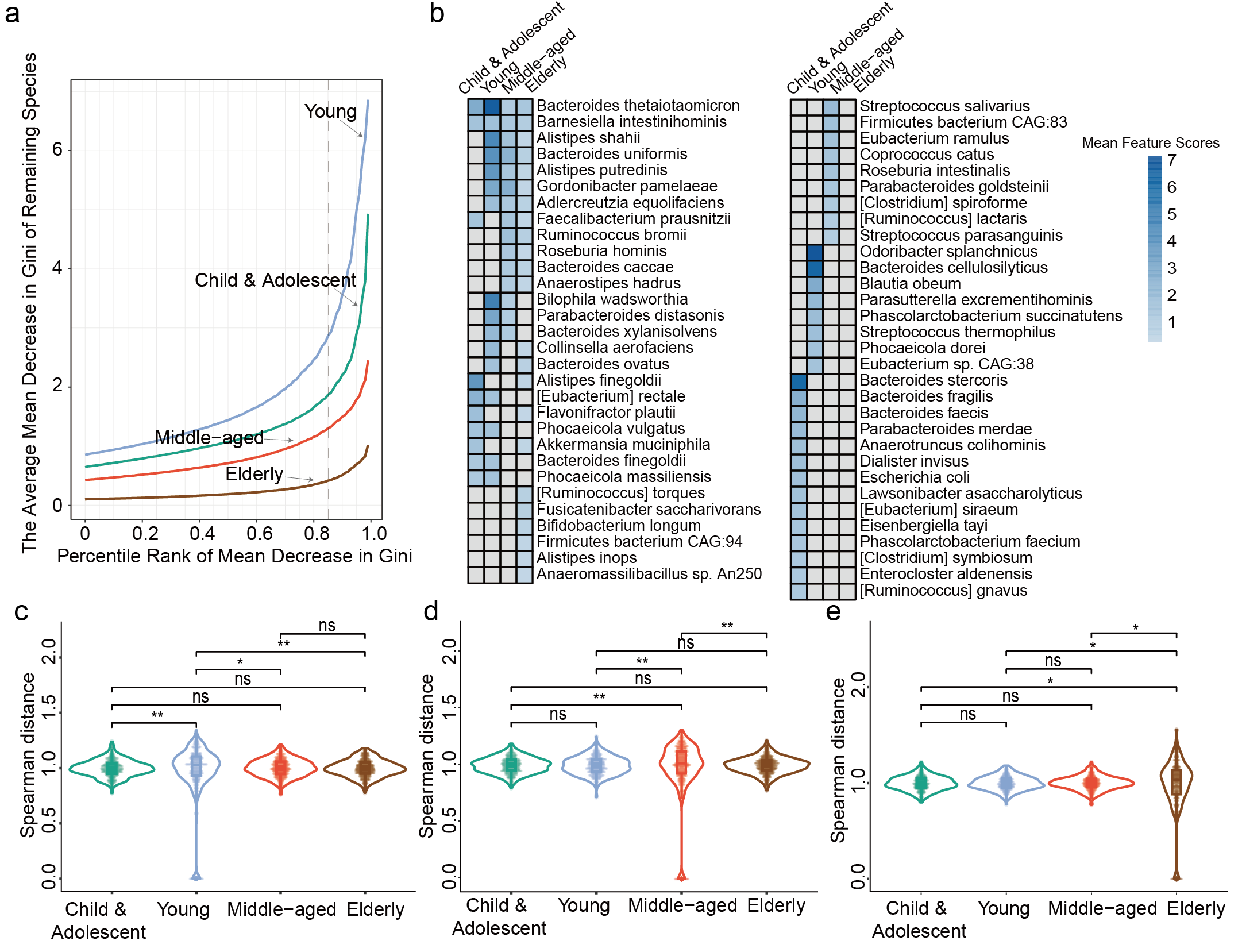


**Figure S7.** Identification of age specificity of the associations between IBD and gut microbiota among women. (a) The fluctuations in mean feature scores of species across percentile ranks. The dotted black lines represented the percentile threshold used to identify IBD species markers for specific age groups. (b) The heatmaps showing the average mean decrease in Gini for IBD age-specific species markers across diverse age groups. The cells in grey signified empty values, indicating that the corresponding species did not serve as an IBD species marker of certain age group. (c-e) The distribution of Spearman distances for IBD age-specific species markers across age groups. IBD markers specific to young adulthood (c), middle-aged adulthood (d) and old age (e) were chosen. Subsequently, Spearman distances were calculated to determine the dissimilarity between the mean feature scores of these markers within the same age group and across other age groups. The comparison of Spearman distances was conducted using Wilcoxon rank sum test. * .01 ≤ FDR < .05; ** .001 ≤ FDR < .01; ns: non-significant.


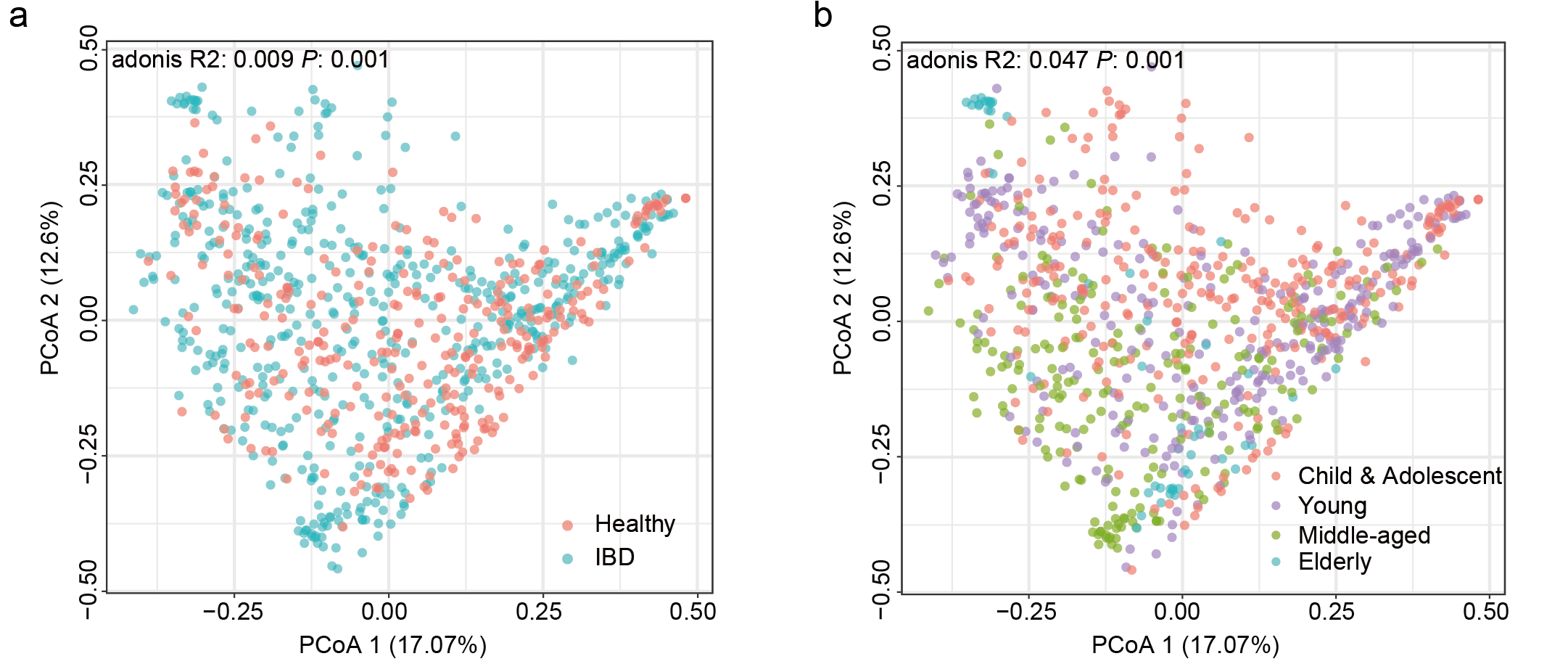


**Figure S8.** Effects of health status (a) and age group (b) on the gut microbiota of IBD sub-cohort. IBD sub-cohort comprised the whole IBD subjects and corresponding healthy controls.
